# The semantic interplay of semantic radicals and host phonograms in chinese transparent character processing

**DOI:** 10.1007/s10339-026-01344-6

**Published:** 2026-04-13

**Authors:** Meng Jiang, Ting Cao, Qi Luo, Jixiang Duan

**Affiliations:** 1 College of Language Intelligence (College of General Education) / Language & Brain Research Center, Sichuan International Studies University, Chongqing, China; 2 School of English Studies, Sichuan International Studies University, Chongqing, China; 3 School of Foreign Languages and Literature, Chongqing Normal University, Chongqing, China

**Keywords:** Semantic radicals, Transparent phonograms, Semantic activation, Semantic interplay

## Abstract

In the realm of Chinese character processing, the semantic activation of semantic radicals and their host phonograms is an intriguing topic. Against the backdrop of previous research which hardly investigated the machinery causing the semantic activation event, the present study is specifically dedicated to addressing this gap. Through an elaborate experiment, employing a priming part of speech judgment task based on the foot semantic radical, the study revealed that the host transparent phonograms incorporating foot semantic radicals demonstrated a significantly greater priming effect than the summation of the priming effect of the host opaque phonograms incorporating foot semantic radicals and that of phonograms which were only foot-related meaning loaded. This unexpected priming enhancement effect suggested a reciprocal interplay between the semantic information of the semantic radicals and that of their host phonograms, introducing a novel source of the semantic information that was engaged in bringing about the semantic activation event*.*

**Supplementary Information:**

The online version contains supplementary material available at 10.1007/s10339-026-01344-6.

## Introduction

Chinese characters generally fall into two categories, namely, pictophonetic compound phonograms and pictographic simple characters (Zhou 1978). The former accounts for approximately 90%, while the latter occupies about 10%. Chinese phonograms are composed of two distinct components: semantic radicals and phonetic radicals (Tan et al. 1995; Zhou et al. 2013). Semantic radicals, typically located on the left side, offer clues to the meaning of the phonograms, while phonetic radicals, usually on the right side, indicate the pronunciation of the phonograms (Chi et al. 2014; Zhang et al. 2014). For instance, in the phonogram “妈” (/ma1/, meaning mother), the component “女” (/nv3/, meaning female) on the left acts as the semantic radical, while “马” (/ma3/, meaning horse) on the right serves as the phonetic radical.

Semantic radicals are categorized into character semantic radicals that can stand alone as simple characters, like “木” (/mu4/, meaning “wood”), and non-character semantic radicals that only function as bound forms within the phonograms, like “氵” (/shui3/, meaning “water”) (Xin 2005; Li 2005).

Phonograms are classified into transparent and opaque types based on the extent of semantic alignment between phonograms and their embedded semantic radicals (Shu & Anderson 1997; Williams & Bever 2010; Zhou et al. 2013). A phonogram is considered transparent when its semantic radical is semantically aligned with the phonogram, as in the phonogram “踢” (/ti1/, meaning “to kick”), where the semantic radical “⻊” is semantically connected to the entire character “踢”. Conversely, it is considered as opaque when there is no such semantic connection, as in the character “捌” (/ba1/, meaning “eight”), where the semantic radical “扌” fails to semantically relate to the whole character “捌”.

### Prior empirical research on the semantic activation of semantic radicals

Of previous studies on Chinese character processing, reams of studies concentrated on examining the semantic activation of semantic radicals, particularly character semantic radicals. Flores d’Arcais et al. (1995), exploiting a priming paradigm and a naming task, found that the semantic information of character semantic radicals was activated, the pre-exposure semantic radical facilitated the naming of characters. Adopting a priming lexical decision task, Ding et al. (2004) found that the pre-exposure of a priming simple character that served as a sub-lexical radical of another target character facilitated the processing of the target, demonstrating the activation of the character semantic radical. Similar effects were reported by Chen and Zhang (2008, 2012), who showed the semantic activation of semantic radicals in the recognition of phonograms. With a primed naming task, Zhou et al. (2013) discovered that character semantic radicals embedded in low-frequency phonograms were semantically activated. Moreover, Zou et al. (2019), using ERPs in a masked priming lexical decision task, observed that character semantic radicals evoked both N400 and Late Positive Complex (LPC), believed to signify semantic activation and extensive processing.

Concurrently, another number of studies examined the semantic activation of non-character semantic radicals in phonogram recognition. For example, Wu et al. (2013) and Wang et al. (2019) provided converging evidence for the semantic activation of the non-character hand semantic radical( e.g., “扌”), with the former showing increased activation in the right medial frontal gyrus during character reading, and the latter demonstrating faster response times when the radical’s semantically implied action direction matched that of the target verb (e.g., “提” [to lift] vs. “捶” [to thump]). Moreover, Hung et al. (2014), used ERPs in a synonym judgment task, presenting character pairs that either shared or did not share the same non-character semantic radical. Results showed that compared with the different semantic radical conditions, synonyms sharing identical semantic radicals elicited reduced M450, while non-synonyms sharing semantic radicals elicited greater M450 especially in the middle frontal region, which suggested the semantic activation of non-character semantic radicals.

In addition, a body of research was committed to investigate the variables modulate semantic activation of semantic radicals- most notably, semantic transparency. For example, Wang et al. (2017), using ERPs techniques, found that compared with the recognition of opaque phonograms, a shorter reaction time, a smaller P200 and a larger N400 were observed for the recognizing of transparent phonograms, indicating that the character semantic radicals incorporated in transparent phonograms were activated more strongly than those incorporated in opaque phonograms. Also, Zhang et al. (2021) investigated how the N170 neural response adapted to sub-lexical semantic and phonological processing by manipulating both the semantic radicals and the semantic relatedness of the four characters that were presented in sequence. The results suggested that N170 exhibited heightened sensitivity to semantic processing, particularly in the right hemisphere during Chinese character reading. Moreover, with a priming paradigm and a lexical decision task, Tong et al. (2021) investigated the priming effect of character semantic radicals, by categorizing the semantic relatedness between the priming semantic radicals and the embedding target phonograms into five levels, and the results showed a graded facilitatory effect on phonogram recognition.

Closely related to semantic transparency is the construct of semantic consistency, a term more commonly used in Chinese psycholinguistic literature to describe the degree of semantic alignment between a semantic radical and its host character. Although terminologically distinct, semantic consistency is conceptually analogous to semantic transparency. In a comprehensive theoretical review, Zhang and Wang (2024) emphasized that semantic consistency is a core determinant of radical-level semantic processing, influencing lexical access, category activation, and semantic feature retrieval across multiple experimental paradigms, including semantic priming tasks, category judgment tasks, feature extraction tasks, and lexical decision paradigms. Wang and Zhang (2016) defined semantic consistency as the degree to which a semantic radical and its host phonogram belong to the same conceptual category and showed that higher consistency facilitated semantic categorization in a word meaning judgment task. Zhang and Zhang (2017) used a priming lexical decision task and found that semantic radicals with higher category consistency elicited stronger semantic activation, as reflected in both accuracy and response time measures.

*Together, these empirical studies converge on the view that semantic radicals are not only semantically activated during phonogram processing but also differentially modulated by properties of the host characters, such as semantic transparency.*

### Theoretical frameworks on the semantic activation of semantic radicals

To understand how semantic radicals contribute to Chinese phonogram recognition, several theoretical models have been proposed to capture the sub-lexical processing mechanisms involved in semantic activation of semantic radicals.

One of the earliest and most influential accounts is the Dual-Network System Model of Chinese character processing, proposed by Zhang and Peng (1993). Drawing on the spreading activation theory (Collins and Loftus 1975) and the principles from the Parallel Distributing Processing (PDF) framework (McClelland and Rumelhart 1981), this model posits two interactive but distinct systems: a lexical network system, which hierarchically processes orthographic and phonological information (from strokes to radicals to morphemes and whole words), and a semantic network system, which stores both conceptual nodes and category-level nodes. Crucially, the model posits semantic radicals serve as intermediary units that can directly activate semantic category nodes in the semantic system, independently of whole character recognition.

Building upon this foundation, Chen and Zhang (2012) proposed the Representation and Processing Model of Semantic Radicals in Lexical Access, which further specifies four hierarchical levels: visual features, semantic radicals, phonograms, and conceptual meanings. The model emphasizes the intermediary role of semantic radicals within the two interconnected systems-lexical and semantic-emphasizing that semantic radical’s function not only as orthographic units but also as category cues that mediate semantic activation. When the meaning of a semantic radical is congruent with that of its host phonogram, lexical access is facilitated. In contrast, when the meaning of the semantic radical is semantically incongruent with the whole character, lexical access may be inhibited.

Furthermore, Wang and Zhang (2016) proposed the Regulatory Model of Semantic Radical Effects on Chinese Phonogram Processing, which provides a more nuanced account of how both family size and category consistency- conceptually akin to semantic transparency modulate the semantic activation of semantic radicals during phonogram recognition. This model posits that within the lexical network system, the ascending pathways of semantic and phonetic radicals, along with the semantic radical-mediated pathway bridging the two systems, play a modulatory role in the phonogram processing. Semantic radicals that belong to a consistent category are more easily semantically activated than those that are semantically inconsistent with their host character. Moreover, category consistency effect on semantic activation of semantic radicals is regulated by family size in error rate.

Finally, the Dual Route Cascaded (DRC) model, proposed by Coltheart et al. (2001), is a computational model of visual word recognition. The model posits that successful word recognition consists of two distinct pathways: the sub-lexical route and the lexical route. In the sub-lexical route, readers sequentially decode the orthographic input using grapheme-to-phoneme correspondence (GPC) rules to generate the phonological representation of the word, then use this phonological representation to activate entries stored in the mental lexicon. In contrast, in lexical route, readers can directly match visually input words to stored orthographic representation in the mental lexicon. Once a match is achieved, the corresponding phonological and semantic representation are activated directly. Although designed for alphabetic scripts, this model’s distinction between holistic and componential processing has been extended to Chinese. Under this view, phonograms composed of semantic and phonetic radicals may engage analogous dual processing pathways: a lexical route for direct character-level recognition and a sub-lexical route for radical-level orthographic recognition.

To recapitulate, an abundance of prior research has arrived at the conclusion that semantic radicals generally undergo semantic activation in their embedding phonograms’ processing, regardless of semantic radicals’ character status. However, three issues remain unresolved. First, prior work has largely established that semantic radicals are activated during phonogram processing, yet it has not decomposed the source and composition of this activation, namely, semantic information at the semantic radical level versus semantic information at the character level, nor clarified how these sources contribute in transparent phonograms. Second, semantic transparency and semantic radical characterhood are seldom orthogonally manipulated within a single, item-matched experiment, making it difficult to isolate their respective and joint contributions, particularly in Chinese character processing. Third, existing studies have predominantly examined character semantic radicals, with comparatively little work on non-character semantic radicals, which limits the generalizability of current accounts.

### The present study

Synthesizing these empirical and theoretical considerations, we posed a central question: what are the distinct sources of semantic information contributing to semantic radical activation in transparent phonograms? Specifically, we addressed three research questions: (1) Did semantic information inherent to the semantic radical contribute to activation in transparent phonograms, independent of the semantic information of the host phonogram? (2) Could semantic information of the whole character alone drive activation, potentially rendering the semantic radical’s own semantic information silent? (3) In transparent phonograms, would the priming effect be greater than the sum of the priming effects attributable to the semantic radical and to the host phonogram, or would it merely equal their sum? To address these questions, the present study aimed to investigate the mechanism that gave rise to the semantic activation of semantic radicals in transparent phonograms. Towards this end, one elaborate experiment was conducted. We employed the priming paradigm, utilizing a part-of-speech judgment task, and manipulated two variables, namely, the prime type and the target type.

Building on the theoretical frameworks reviewed above, converging accounts suggest that semantic information is accessible at both the radical and whole-character levels and that alignment between them (semantic transparency/consistency) tends to strengthen semantic activation; accordingly, we contrast an interactive account with a conservative additive view that treats the two sources as independent. Under the Dual-Network System (Zhang and Peng 1993), the Representation-and-Processing Model of Semantic Radicals (Chen and Zhang 2012), and the Regulatory Model of Semantic Radical Effects (Wang and Zhang 2016), alignment is expected to yield convergent semantic activation, whereas the DRC model predicts at most additive contributions (Coltheart et al. 2001). These considerations lead to three predictions aligned with our three questions. First, if semantic information inherent to the semantic radical contributes to semantic activation, opaque phonograms containing the *foot* semantic radical should show a significant priming effect, that is, shorter reaction times for targets preceded by foot primes than for those preceded by control primes. Second, if host character’s semantic information alone can drive semantic activation, foot meaning-loaded only characters (without the *foot* semantic radical) should also show the same pattern. Third, if transparent phonograms engage interactive processing, then transparent phonograms containing the *foot* semantic radical should produce enhanced priming that exceeds simple additivity, namely, the magnitude of priming effect would be greater than the sum of the priming effects attributable to the radical-level source and to character-level source.

## Method

### Participants

A prior sample size was calculated using G*Power 3.1 (Faul et al. 2007) to estimate the required sample size. To detect a medium effect size (f = 0.25) (Cohen 1969) with 80% power for a two-way within-subjects ANOVA (α = 0.05, number of groups = 1, number of measurements = 2*5 = 10, non-sphericity correction = 1), the analysis suggested that at least 22 participants would be required. These calculations were conducted in accordance with the guidelines for repeated measures within-subjects ANOVA (a priori) presented in the G*Power 3.1 Manual (2023) and Brysbaert (2019). To ensure the validity of the data, possible exclusions were considered. A total of 34 participants (4 male, 30 female) were recruited in the experiment who were native Mandarin speakers living in mainland China (mean age = 25 years, SD = 2.90). All participants were right-handed students from Sichuan International Studies University (SISU), possessing normal or corrected-to-normal vision. They were required to provide written informed consent before the experiment and received compensation after the experiment.

#### Materials and design

The experiment adopted a 2 (*Prime Type: foot picture vs. control picture*) × 5 (*Target Type: transparent phonograms embedding character foot semantic radical vs. transparent phonograms embedding non-character foot semantic radical vs. foot meaning-loaded only characters vs. opaque phonograms embedding character foot semantic radical vs. opaque phonograms embedding non-character foot semantic radical*) design. These five levels of Target Type reflected different combinations of two conceptual dimensions: the characterhood of the semantic radical (character vs. non-character) and the semantic transparency between the radical and its host phonogram (transparent vs. opaque).

The prime type included a human foot picture, and a control picture which was made by distorting the human foot picture through the utilization of the software GIMP, such that it was no longer discernible.

As for the target characters, the first type was transparent phonograms embedding the character *foot* semantic radicals that took on meaning related to human foot effector (e.g., the target phonogram “跃” /yue4/, meaning “to jump”, contains the character *foot* semantic radical “”, and denotes the meaning of bodily motion executed by human foot effector). The second type was the same as the first one except that the semantic radicals embedded therein were non-character *foot* ones (e.g., the target phonogram “逛” (/guang4/, meaning “to stroll”, contains the non-character *foot* semantic radical “辶” /chuo4/, and denotes the meaning of human foot effector-executed motion). The third type was *foot* meaning-loaded only characters (e.g., the target phonogram “舞” /wu3/, meaning “to dance”, contains no *foot* semantic radicals but still denotes the human foot effector-executed motion meaning). The fourth type was opaque phonograms embedding the character *foot* semantic radicals (e.g., the target phonogram “跎” /tuo2/, meaning “to idle”, contains the character *foot* semantic radical “”, but denotes no meaning relevant to the bodily motion executed by human foot effector). The fifth type was the same as the fourth one except that the semantic radicals embedded therein were non-character ones (e.g., the target phonogram “逼” /bi1/, meaning “to compel”, contains the non-character *foot* semantic radical “辶” and denotes no meaning relevant to the bodily motion executed by human foot effector).

To validate the transparency classification of the target characters, we conducted a semantic transparency rating task with 20 native Chinese speakers (none of whom took part in the main experiment). In line with the theoretical notions of semantic transparency and semantic consistency introduced in the Introduction, we operationally defined semantic transparency as the degree of semantic connection or correlation between the semantic radical and the whole character’s meaning. Participants were asked to rate this connection on a 7-point Likert scale (1 = completely unrelated, 7 = highly related). The mean semantic transparency ratings of the first two types of characters were 6.42 (SD = 0.13) and 6.36 (SD = 0.27) respectively. The mean semantic transparency of the third type of characters was 5.28 (SD = 0.24), even without explicit *foot* semantic radicals, likely because of their strong association with human foot-executed motion semantics. The mean semantic transparency ratings of the last two types of characters were 2.97 (SD = 0.68) and 2.84 (SD = 0.43) respectively. Paired sample t-test between the mean semantic transparency of the first two types (*t*(9) = 0.57, *p* = 0.580) and the last two types of characters (*t*(9) = 0.57, *p* = 0.585) showed no difference. However, a paired sample t-test between the transparent and opaque conditions demonstrated significant differences (*t*(19) = 27.66, *p* < 0.001).

Each type consisted of 10 Chinese characters (see Table 1 for sample materials). In the same order as the five target types defined above, the mean number of strokes was 13.80 (SD = 1.62), 11.90 (SD = 2.03), 11.90 (SD = 1.37), 14.00 (SD = 2.98), and 12.10 (SD = 2.03); the mean frequency (raw counts) was 83,697.60 (SD = 94,822.16), 98,886.20 (SD = 107,267.89), 101,584.10 (SD = 123,156.58), 7,321.10 (SD = 7,450.80), and 150,627.80 (SD = 145,485.58). The one-way ANOVA indicated no significant differences across types in the number of strokes (*F*(4,36) = 2.58, *p* = 0.053) or mean frequency (*F*(4,36) = 1.98, *p* = 0.157). All targets were verbs. The frequency of each target character was obtained from the corpus compiled by the Beijing Language and Culture University (BLCU) Corpus Center, which is widely used in Chinese psycholinguistic research.

**Table 1:**
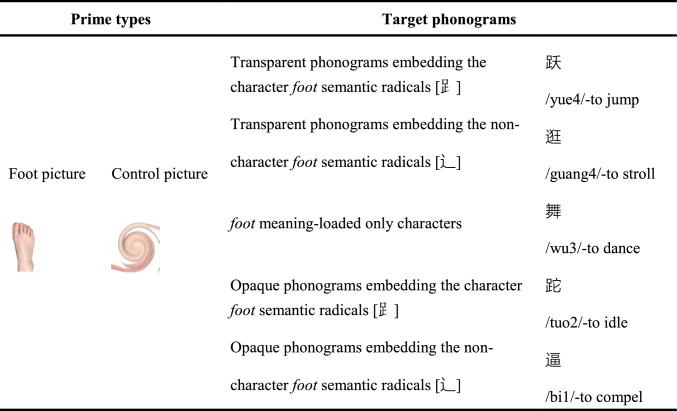
Sample materials and their properties in the experiment

To support the priming part-of-speech judgment task, 20 nouns and adjectives were selected as fillers (see the Supplementary Materials for details). The means for the number of strokes, frequency and familiarity of these two filler types were as follows: for stroke, 9.45 (SD = 1.99), 10.50 (SD = 2.33); for frequency (raw counts), 656,597.00 (SD = 671,857.31), 435,171.45 (SD = 639,978.33); for familiarity, 6.80 (SD = 0.04), 6.80 (SD = 0.03). These two filler types were matched on the number of strokes (*t*(19) = -1.69,* p* = 0.108), mean frequency (*t*(19) = 1.29, *p* = 0.214) and familiarity (*t*(19) = -0.24, *p* = 0.815). Familiarity ratings were collected from 20 native Chinese speakers (who did not participate in the main experiment) using a 7-point Likert scale, to ensure that there were no significant differences in familiarity across the five types of target characters and the two types of fillers. The mean familiarity for five types was 6.67 (SD = 0.12), 6.59 (SD = 0.17), 6.66 (SD = 0.14), 6.58 (SD = 0.18) and 6.70 (SD = 0.08), with one-way ANOVA indicating no significant differences across the five target types (*F*(4,36) = 1.26, *p* = 0.303). Frequency data for fillers were also sourced from the BLCU corpus. Two types of prime pictures were matched with 50 target characters and 20 controls, with a total of 140 prime-target pairs being fully used across trials.

#### Procedure

Participants were seated approximately 50 cm from a computer in a comfortable chair. Each trial began with a fixation signal for 300 ms, followed by a blank screen for 300 ms. The prime picture was then presented for 500 ms, followed by another 300 ms blank screen. The target (a phonogram, either a verb or a noun) was presented until the participants had made a response. The next trial began after a random interval of 600 to 800 ms. Participants were instructed to determine whether the target character was a verb or not as quickly as possible by pressing the “F” or “J” keys. The configuration for “pressing key” were counterbalanced across participants, with half using “F” for “NOUN” and “J” for “VERB”, and the other half vice versa. Accuracy and reaction times were recorded. This experiment was conducted in the Key Laboratory of Foreign Language Learning and Cognitive Neuroscience in SISU.

## Results

Data from participants whose accuracy on the part-of-speech judgment task fell below 90% were excluded from analysis, given the relative simplicity of the task. As a result, data of 30 participants were included in the statistical analysis. The overall mean accuracy for the part-of-speech judgment task was 96.53%. The mean accuracies by target type were: transparent phonograms embedding the character *foot* semantic radical, 96.17% (SD = 5.70%); transparent phonograms embedding the non-character *foot* semantic radical, 96.83% (SD = 4.51%); foot meaning-only characters, 97.25% (SD = 3.84%); opaque phonograms embedding the character *foot* semantic radical, 96.50% (SD = 4.98%); and opaque phonograms embedding the non-character *foot* semantic radical, 95.92% (SD = 4.65%). Error trials and trials with reaction time (RT) exceeding 3 SD from the mean RT (9.78%) were also excluded from further analyses. Table 2 displays the mean reaction times and mean accuracy.

**Table 2:** Mean RT (ms) and Accuracy (%) for part-of-speech judgment in the experiment

Prime type	Target characters				
	Transparent phonograms embedding the character *foot* semantic radicals	Transparent phonogram embedding the non-character *foot* semantic radicals	*Foot* meaning-load-ed only characters	Opaque phonograms embedding the character *foot* semantic radicals	Opaque phonograms embedding the non-character *foot* semantic radicals
RT					
Foot	564 (78)	619 (84)	658 (111)	653 (133)	703 (117)
Control	632 (84)	689 (102)	679 (111)	676 (133)	718 (124)
Difference Value	68	70	21	23	15
Accuracy					
Foot	95.00(6.82)	97.00(4.07)	96.50(4.58)	95.83(5.27)	95.50(4.97)
Control	97.33(4.10)	96.67(4.97)	98.00(2.82)	97.17(4.68)	96.33(4.34)
Mean	96.17(5.70)	96.83(4.51)	97.25(3.84)	96.50(4.98)	95.92(4.65)

*Note*. Values are means with standard deviations in parentheses (SD)

A two-way repeated-measures ANOVA was performed on the RTs. Results showed that the main effect of Prime Type was significant (*F* (1, 29) = 64.26, *p* < 0.001, MSE = 115,797.45, η^2^_p_ = 0.69). The main effect of Target Type was also significant (*F* (3.18, 92.16) = 18.82, *p* < 0.001, MSE = 123,023.39, η^2^_p_ = 0.39). The interaction effect between Prime Type and Target Type was also significant (*F* (3.049, 88.42) = 26.79, *p* < 0.001, MSE = 14,350.54, η^2^_p_ = 0.48).

The post hoc analysis of the main effect of Prime type showed that RTs to target characters preceded by foot picture (639 ms) were significantly faster than those preceded by control picture (679 ms) (*p* < 0.001). The post hoc analysis of the main effect of Target character demonstrated that RTs to transparent targets with character foot semantic radicals (598 ms) were the fastest, followed by the RTs to the foot meaning-loaded only characters (654 ms), the RTs to transparent targets with non-character foot semantic radicals (665 ms), and the RTs to opaque targets embedding the non-character foot semantic radicals (669 ms). RTs to the opaque phonograms embedding the character foot semantic radicals were the slowest (710 ms).

To further assess the magnitude of priming effects, paired-samples t-tests were conducted, comparing the size of priming effects (defined as the RT difference between control-picture and foot-picture conditions) across five types. As mentioned in the introduction, we hypothesized that semantic activation of semantic radicals may arise from three potential sources: semantic information of the semantic radicals themselves, semantic information of the whole character, and the interplay of the semantic information from both semantic radicals and their embedding characters. According to this rationale, we tested whether the priming effects for transparent phonograms embedding *foot* semantic radical exceeded the combined effects of foot meaning-loaded only characters (which reflects the semantic information of the whole character) and opaque phonograms with *foot* semantic radical (which reflects the semantic information of the semantic radicals).

The transparent target phonograms embedding the character *foot* semantic radicals exhibited significantly larger priming effects (632 ms _RT-control picture_–564 ms _RT-*foot* picture_ = 68 ms) than the summation (43 ms) of the priming effects of the *foot* meaning-loaded only characters (679 ms _RT-control picture_–658 ms _RT-*foot* picture_ = 21 ms) and that of the opaque target phonograms embedding the character *foot* semantic radicals (676 ms _RT-control picture_–653 ms _RT-*foot* picture_ = 23 ms) (*t*(30) = 5.78, *p* < 0.001, Cohen’s d = 1.01).

Likewise, the transparent target phonograms embedding the non-character *foot* semantic radicals (689 ms _RT-control picture_–619 ms _RT-*foot* picture_ = 70 ms) exhibited significantly larger priming effects than the summation (36 ms) of the priming effects of the *foot* meaning-loaded only characters (679 ms _RT-control picture_–658 ms _RT-*foot* picture_ = 21 ms) and that of the opaque target phonograms embedding the non-character *foot* semantic radicals (718 ms _RT-control picture_–703 ms _RT-*foot* picture_ = 15 ms) (*t*(30) = 6.50,* p* < 0.001, Cohen’s d = 1.19).

## Discussion

The semantic activation of semantic radicals is of great concern in Chinese character processing. However, no thorough inquiry has been made into the cause of the sub-lexical semantic activation event. How does this event arise? Is it merely the retrieval of a semantic radical’s own meaning, or does the host character also contribute, and do these sources interact? Our results showed that transparent target phonograms containing either character or non-character *foot* semantic radicals (e.g., “跃”/yue4/, meaning “to jump”; “逛”/guang4/, meaning “to stroll”) exhibited a priming benefit that exceeded both the character-level component (approximated by the *foot* meaning-loaded only character, e.g., “舞”/wu3/, meaning “to dance”) and the radical-level component (approximated by the opaque target phonograms embedding* foot* semantic radicals, e.g., “跎”/tuo2/, meaning “to idle”; “逼”/bi1/, meaning “to compel”). It was actually larger than the summation of the two.

First, this finding reinforces the point that the semantic radical is semantically activated during Chinese phonogram recognition. This is consistent with prior evidence from Flores d’ Arcais, Saito, and Kawakami (1995), Ding et al. (2004), and Zou et al. (2019), who reported the priming effect induced by the semantic radicals. Our findings support core claims of most psycholinguistic models of character recognition that highlight the role of sub-lexical components during Chinese character recognition. For example, the Dual-Network System Model (Zhang and Peng 1993) postulates that semantic radicals operate within a semantic network parallel to the lexical network and can independently activate semantic category nodes even before whole-character recognition is completed. This early access to semantic categories may underline the observed priming effects.

Moreover, our finding that transparent phonograms elicited robust priming effects resonates with Tong et al. (2021), who found a graded priming effect of the semantic radical on Chinese character identification as a function of semantic relatedness between semantic radicals and their host phonograms. Importantly, the present study goes beyond previous findings by demonstrating that this priming effect is not merely the simple summation, but reflects an enhanced interplay between semantic information of semantic radicals and that of the whole characters.

Regarding the transparent target phonograms which embedded both character and non-character *foot* semantic radicals, we expected that the priming effects would be the combination of the priming effects engendered by the meaning of the host phonograms and that engendered by the meaning of the *foot* semantic radicals. The former was equivalent to the priming effect that occurred to the *foot* meaning-loaded only characters, while the latter amounted to that occurring to the *foot* semantic radicals embedded in the opaque target phonograms. As such, the magnitude of the priming effects of the transparent target phonograms which embedded the *foot* semantic radicals should be as large as the combined priming effects engendered both by the semantic information of the whole phonograms and by the semantic information of the *foot* semantic radicals. However, it significantly exceeded the combination of those two sources of priming effects. What accounts for this unexpected discrepancy? Where does the surplus priming effect come from? Given the experimental design wherein both the embedded *foot* semantic radicals and their host phonograms had close semantic connection with the *foot* picture prime, the enhanced priming effects could be preferably ascribed to the interaction of the above-mentioned two sources of the priming effects.

From a theoretical perspective, this effect is well explained by models that emphasize dynamic interaction between sub-lexical and lexical components during character recognition. The Regulatory Model of Semantic Radical effect on Phonogram Processing (Wang and Zhang 2016) proposes that the activation of semantic radicals is modulated by both family size and category consistency. In the case of transparent phonograms, where the semantic radical and the host character belong to the same semantic category, the radical-mediated route between the lexical system and the semantic system is more easily activated. This congruence facilitates stronger and more efficient semantic access, contributing to the enhanced priming effects observed in the current study.

Taken together, the present study identified a very novel finding. Research attention has conventionally been directed to the characterization of semantic activation of the semantic radical as a surface manifestation. No research efforts have been made to examine the underlying mechanism that enables this semantic activation event. It has generally been taken for granted that the semantic activation of the semantic radical is no more than a matter of retrieval of the sub-lexical units’ own semantic information. The present study, however, revealed that the semantic radicals’ semantic information and the host phonograms’ semantic information executed an interplay with each other, jointly giving rise to a new source of semantic information, adding silently to the semantic radical’s semantic activation. In short, there might exist an interplay mechanism that governs the semantic activation of semantic radicals. The semantic activation behavior of the semantic radicals may mean a more complicated story than it appears.

## Conclusion

In the realm of Chinese character processing studies, the focal attention was given to the presence of the semantic activation of semantic radicals. Little attention was paid to the underlying mechanism that made the activation event possible. The present study, by recruiting transparent phonograms built from the Chinese character and non-character *foot* semantic radicals in the experiment, probed into the on-goings behind that might have brought about the semantic radical’s semantic activation event. It was found that the semantic activation of semantic radicals was a matter of two activation events: the activation of the sub-lexical unit’s own semantic information, and the interactive activation of both the sub-lexical unit’s semantic information and that of the host phonograms. This was a rather novel finding, which may throw light on a deeper understanding of semantic radicals’ semantic activation event. Future studies should further investigate this interplay using neurocognitive methodologies such as ERP and fMRI, and extend the inquiry to other commonly used semantic radicals (e.g. “扌”, “氵”) to examine the generalizability of these findings.

## Supplementary Information

Below is the link to the electronic supplementary material.

Supplementary file1 (DOCX 30 KB)

## Data Availability

The data that support the findings of this study are openly available in OSF at https://osf.io/msz3f. The datasets and materials generated during the current study are available from the corresponding author on reasonable request.
