# Supplementary material for: The semantic interplay of semantic radicals and host phonograms in chinese transparent character processing
Source: Cogn Process. 2026 Apr 13;27(3):565–73. doi: 10.1007/s10339-026-01344-6 (PMC13424633; doi:10.1007/s10339-026-01344-6)
Supplement: Supplementary file 1 — Supplementary file1 (DOCX 30 KB) [file 10339_2026_1344_MOESM1_ESM.docx]

# Supplementary Materials

Target phonograms used in the experiment

| Item No. | Transparent phonograms embedding the character *foot* semantic radicals | Transparent phonograms embedding the non-character *foot* semantic radicals | *Foot* meaning-loaded only phonograms | Opaque phonograms embedding the character *foot* semantic radicals | Opaque phonograms embedding the non-character *foot* semantic radicals |
| --- | --- | --- | --- | --- | --- |
| 1 | 跳/tiao4/ | 遣/qian3/ | 登/deng1/ | 跎/tuo2/ | 遭/zao1/ |
| 2 | 跨/kua4/ | 逾/yu2/ | 躲/duo3/ | 趴/pa1/ | 遍/bian4/ |
| 3 | 踩/cai3/ | 逐/zhu2/ | 徘/pai2/ | 躇/chu2/ | 造/zao4/ |
| 4 | 跑/pao3/ | 遨/ao2/ | 溜/liu1/ | 蹉/cuo1/ | 逗/dou4/ |
| 5 | 踹/chuai4/ | 遁/dun4/ | 窜/cuan4/ | 踰/yu2/ | 逮/dai3/ |
| 6 | 跃/yue4/ | 逛/guang4/ | 滚/gun3/ | 踌/chou2/ | 逼/bi1/ |
| 7 | 踮/dian3/ | 避/bi4/ | 舞/wu3/ | 跻/ji1/ | 遗/yi2/ |
| 8 | 跺/duo4/ | 遛/liu1/ | 站/zhan4/ | 跛/bo3/ | 遮/zhe1/ |
| 9 | 踏/ta1/ | 退/tui4/ | 驰/chi2/ | 踊/yong3/ | 邀/yao1/ |
| 10 | 踢/ti1/ | 遇/yu4/ | 骋/cheng3/ | 蹭/ceng4/ | 逝/shi4/ |

Fillers (i.e. nouns and adjectives) used in the experiment.

| Item No. | Nouns | Adjectives |
| --- | --- | --- |
| 1 | 朋/peng2/ | 胖/pang4/ |
| 2 | 框/kuang1/ | 粗/cu1/ |
| 3 | 姐/jie3/ | 辣/la4/ |
| 4 | 树/shu4/ | 烦/fan2/ |
| 5 | 纸/zhi3/ | 酸/suan1/ |
| 6 | 财/cai2/ | 稳/wen3/ |
| 7 | 碳/tan4/ | 温/wen1/ |
| 8 | 线/xian4/ | 强/qiang2/ |
| 9 | 袜/wa4/ | 长/chang2/ |
| 10 | 根/gen1/ | 净/jing4/ |
| 11 | 酒/jiu3/ | 矮/ai3/ |
| 12 | 瓶/ping2/ | 细/xi4/ |
| 13 | 妹/mei4/ | 冷/leng3/ |
| 14 | 枝/zhi1/ | 倦/juan4/ |
| 15 | 球/qiu2/ | 甜/tian2/ |
| 16 | 海/hai3/ | 静/jing4/ |
| 17 | 境/jing4/ | 暖/nuan3/ |
| 18 | 杆/gan1/ | 轻/qing1/ |
| 19 | 钱/qian2/ | 脆/cui4/ |
| 20 | 涛/tao1/ | 脏/zang1/ |
